# Research on Drive and Detection Technology of CMUT Multi-Array Transducers Based on MEMS Technology

**DOI:** 10.3390/mi16060604

**Published:** 2025-05-22

**Authors:** Chenyuan Li, Jiagen Chen, Chengwei Liu, Yao Xie, Yangyang Cui, Shiwang Zhang, Zhikang Li, Libo Zhao, Guoxing Chen, Shaochong Wei, Yu Gao, Linxi Dong

**Affiliations:** 1 College of Electronics and Information, Hangzhou Dianzi University, Hangzhou 310018, China; 222040300@hdu.edu.cn (C.L.); 2343040033@hdu.edu.cn (J.C.); 241040028@hdu.edu.cn (Y.C.); 2 Suzhou Thermal Power Research Institute Co., Ltd., Suzhou 215004, China; liuchengwei@cgnpc.com.cn (C.L.); yaoxie1201@outlook.com (Y.X.); chenguoxing@cgnpc.com.cn (G.C.); weishaochong@cgnpc.com.cn (S.W.); 3 State Key Laboratory for Manufacturing Systems Engineering, State Industry-Education Integration Center for Medical Innovations, International Joint Laboratory for Micro/Nano Manufacturing and Measurement Technologies, Shaanxi Innovation Center for Special Sensing and Testing Technology in Extreme Environments, Shaanxi Provincial University Engineering Research Center for Micro/Nano Acoustic Devices and Intelligent Systems, Xi’an Jiaotong University, Xi’an 710049, China; 3122301008@stu.xjtu.edu.cn (S.Z.); zhikangli@xjtu.edu.cn (Z.L.); libozhao@xjtu.edu.cn (L.Z.)

**Keywords:** micro-electro-mechanical systems (MEMS), capacitive micromachined ultrasonic transducer (CMUT), finite element analysis (FEA), driving and detection

## Abstract

This paper presents an ultrasonic driving and detection system based on a CMUT array using MEMS technology. Among them, the core component CMUT array is composed of 8 × 8 CMUT array elements, and each CMUT array element contains 6 × 6 CMUT units. The collapse voltage of a single CMUT unit obtained through finite element analysis is 95.91 V, and the resonant frequency is 3.16 MHz. The driving section achieves 64-channel synchronous driving, with key parameters including an adjustable excitation signal frequency ranging from 10 kHz to 5.71 MHz, a delay precision of up to 1 ns, and an excitation duration of eight pulse cycles. For the echo reception, a two-stage amplification circuit for high-frequency weak echoes with 32 channels was designed, achieving a gain of 113.72 dB and −3 dB bandwidth of 3.89 MHz. Simultaneously, a 32-channel analog-to-digital conversion based on a self-calibration algorithm was implemented, with a sampling rate of 50 Mbps and a data width of 10 bits. Finally, the experimental results confirm the successful implementation of the driving system’s designed functions, yielding a center frequency of 1.4995 MHz and a relative bandwidth of 127.9%@−6 dB for the CMUT operating in silicone oil. This paper successfully conducted the transmit–receive integrated experiment of the CMUT and applied Butterworth filtering to the echo data, resulting in high-quality ultrasonic echo signals that validate the applicability of the designed CMUT-based system for ultrasonic imaging.

## 1. Introduction

Ultrasonic technology, with its significant advantages of being radiation-free, offering high resolution, and enabling real-time detection, has become an indispensable tool in modern testing fields [1]. The ultrasonic transducer, as a key component of ultrasonic systems, directly affects the detection performance with its quality. Since the late 20th century, the breakthrough development of micro-electro-mechanical systems (MEMS) technology has spurred innovations in micromechanical ultrasonic transducers, giving rise to both capacitive micromachined ultrasonic transducers (CMUTs) and piezoelectric micromachined ultrasonic transducers (PMUTs) [2,3].

As emerging mainstream ultrasonic transducers, PMUTs and CMUTs are suited to different application scenarios due to differences in their working principles and performance characteristics [4]. Due to their low driving voltage requirements, PMUTs are particularly advantageous for wearable real-time monitoring applications [5]. Hong Ding et al. developed a wearable blood flow monitoring system utilizing AlN-based PMUT probes integrated with a pulsed wave Doppler (PWD) technique. The system incorporates a linear fitting calibration process to enhance the accuracy of blood flow measurements [6]. Compared to PMUTs, CMUTs have become a forefront research direction due to their wider bandwidth, higher sensitivity, and unique potential for miniaturization and integration [7]. Moreover, CMUTs offer low fabrication cost and are compatible with flip-chip bonding for monolithic integration on ASICs. Additionally, they can be easily arranged into arrays, enabling 3D ultrasonic imaging [8]. Currently, CMUTs are widely used in various fields such as ultrasound imaging [9,10], high-intensity focused ultrasound (HIFU) [11,12], and photoacoustic imaging (PAI) [13,14].

Considering the current manufacturing technology and CMUT performance, CMUT microelements are mainly designed in three shapes: rectangular [15], hexagonal [16], and circular [17]. Xiao Huang and Hongliang Wang, through theoretical analysis and finite element simulations of CMUTs, compared three different CMUT structures. The results indicate that although the rectangular CMUT exhibits the largest membrane deflection and higher acoustic transmission efficiency [18], the circular CMUT, with its minimal edge stress, highest stability, and transmission performance meeting signal detection requirements, shows a clear advantage among the three structures [19]. The main structural parameters of CMUTs include radius, cavity height, and membrane thickness. Zhikang Li and Libo Zhao derived the membrane deflection, collapse voltage, and resonant frequency of CMUTs under conditions of up to 96% collapse voltage, diameter-to-thickness ratios of 20–80, and gap-to-thickness ratios of ≤2. Their calculated results are consistent with simulations, providing important guidance for CMUT design [20].

CMUT array elements are composed of various CMUT units, and these elements are arranged in a specific pattern to form an array design. By driving the CMUT array elements at different time points, ultrasonic beam steering and focusing effects can be achieved. Yu Pei and Yu Zhang et al. proposed a one-dimensional CMUT array comprising 128 array elements, each containing 600 CMUT units. Each element measures 10 mm in length and 0.65 mm in width, with an inter-element spacing of 0.35 mm and an area of 134.5 mm^2^. The array operates at a central frequency of 3.005 MHz with a relative bandwidth of 111.7%@−6 dB, and its imaging feasibility was validated through simulation and breast phantom experiments [21]. The sensor has further optimization potential in terms of volume design and array structure. The two-dimensional sensor demonstrates relatively better imaging performance. Oluwafemi J. Adelegan and Zachary A. Coutant, along with colleagues, proposed a two-dimensional CMUT array comprising 16 × 16 array elements, with each element consisting of 3 × 3 CMUT units. They fabricated the device using a sacrificial release method on an alkali-free glass substrate. Test results showed that the center operating frequency was 4.76 MHz and the average capacitance per array element was 1.17 pF [22]; this design can improve signal detection quality by reducing the number of array elements while increasing the number of CMUT units per element.

From the aforementioned CMUT array designs, it is evident that most existing implementations adopt one-dimensional linear arrays or large-scale arrays with over 100 channels. However, these designs often neglect key practical challenges such as signal strength degradation and increased hardware complexity. Currently, CMUT drive and detection are mostly implemented using discrete components such as pulse generators and oscilloscopes, making it difficult to drive multi-array CMUTs and achieve precise phase control [23,24]. Omid Farhanieh and Ali Sahaf et al. designed an intravascular HIFU drive chip based on 0.35 µm high-voltage CMOS technology (1.85 × 1.8 mm^2^), integrating eight high-voltage drivers (20 Vpp/10 MHz, 67 mW/channel), a digital beamformer (11.25° phase accuracy), and a tunable clock source. This provides a solution for intravascular ultrasound driving, such as CMUT-based minimally invasive tumor ablation [25]. Meiyi Zhou and Sotir Ouzounov et al. designed a receive analog front-end (RX AFE) system for ultrasound harmonic imaging. The system integrates a programmable 2 MHz–15 MHz band-pass filter, a two-stage self-biased inverter-based transimpedance amplifier (TIA), and an 8-bit 80 MS/s analog-to-digital converter (ADC). Experimental results demonstrate that the system maintains low power consumption while providing reliable hardware support for high-resolution ultrasound imaging applications [26].

Building upon a modular hardware architecture, this study presents an innovative integration of CMUT driving and receiving functionalities. The transmission module supports multi-channel synchronized phase control with high-precision algorithms, while the receiving module enables self-calibrated signal acquisition. This design is effectively tailored to the developed 64-channel CMUT chip.

## 2. Design and Implementation of CMUTs

### 2.1. Theoretical Analysis

#### 2.1.1. Structural Description

**Figure 1:**
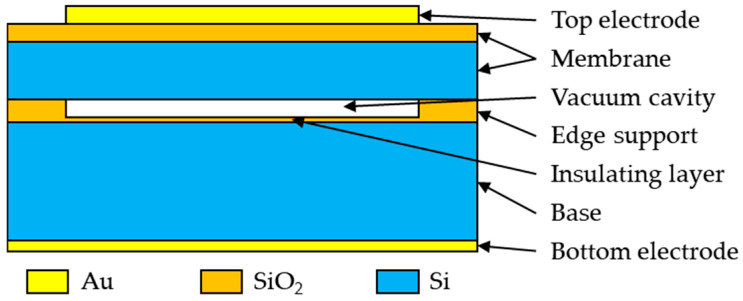
Diagram of a circular CMUT cell structure.

A CMUT array is composed of CMUT cells. Thus, the performance of CMUT cells will directly determine the performance of the CMUT array. The CMUT cell features a typical capacitive structure, primarily composed of a top electrode, a vibrating membrane, a vacuum cavity, an edge support ring, an insulating layer, a substrate, and a bottom electrode [27], as shown in Figure 1. The structural design parameters of the CMUT cells are detailed in Table 1.

**Figure 2:**
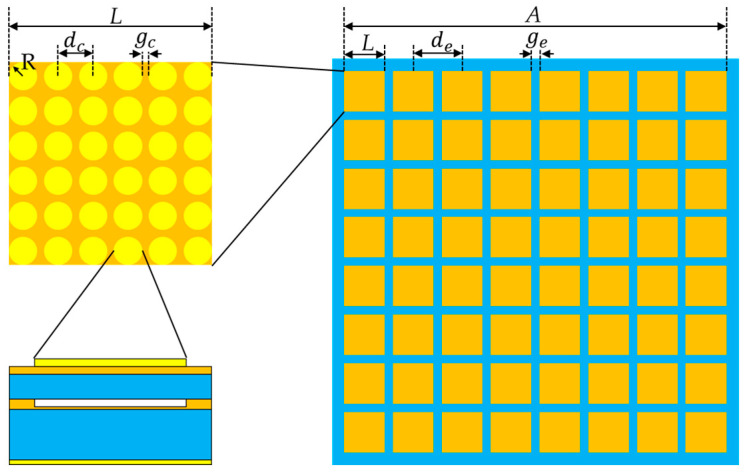
Diagram of the CMUT array structure.

The design of the CMUT array should conform to the principles of maximizing the main lobe strength (to enhance the echo signal amplitude), minimizing the main lobe width (to improve spatial resolution), eliminating grating lobes (to avoid aliasing artifacts), and suppressing side lobes (to reduce background noise). In this study, the proposed CMUT array is constructed using an 8 × 8 configuration of CMUT elements, with each element further composed of a 6 × 6 matrix of CMUT cells, as shown in Figure 2. This hierarchical structure enables the aggregation of multiple CMUT cells into one functional unit, effectively boosting the output signal strength per channel while reducing the complexity of system-level hardware control. After comprehensively considering the structural design and the effect of mutual radiation impedance on CMUT performance [28], the spacing between CMUT array elements and CMUT unit elements was determined, as shown in Table 2.

**Figure 3:**
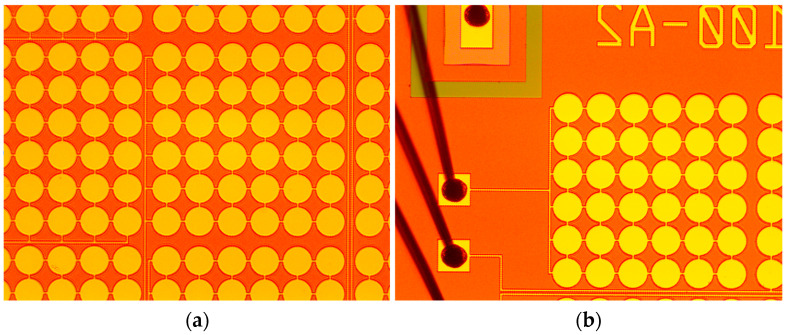
Detailed view of the CMUT array. (**a**) Main view of the CMUT array. (**b**) Wire bonding diagram of the CMUT array.

The CMUT array was fabricated using a high-temperature wafer direct bonding process. The fabrication of the array was finally completed, as shown in Figure 3.

#### 2.1.2. Operating Modes

**Figure 4:**
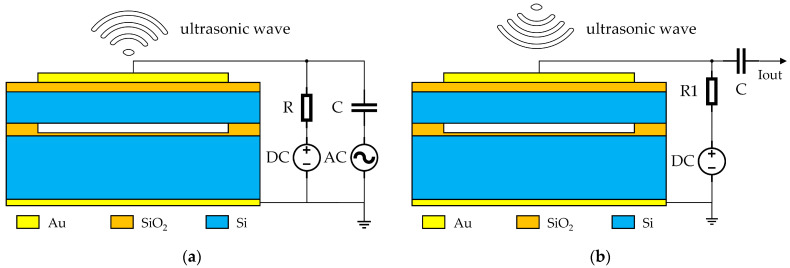
CMUT operating modes. (**a**) Transmission mode. (**b**) Reception mode.

The operating principle of CMUT is based on the coupling of electrostatic forces and membrane vibration, enabling bidirectional conversion between acoustic and electrical energy. It operates in two modes, transmission and reception, as shown in Figure 4. In transmission mode, an AC excitation signal is applied, causing the membrane to vibrate at high frequency under alternating electrostatic forces, thereby compressing or expanding the cavity and radiating ultrasonic waves into the medium. In reception mode, incoming ultrasonic waves make the membrane vibrate, causing CMUT capacitance variations detected as current fluctuations to extract acoustic information. A DC bias voltage is applied in both modes to boost transmission efficiency and reception sensitivity.

#### 2.1.3. Characteristic Parameters

The operation of CMUT is driven by the combined effect of a bias voltage and an AC excitation signal, with two key parameters: collapse voltage and resonant frequency. The collapse voltage refers to the critical bias voltage at which the CMUT membrane undergoes irreversible deformation. As the bias voltage approaches this limit, the electrostatic attraction between the electrodes increases rapidly, causing the membrane-to-electrode gap to shrink significantly. Once this threshold is exceeded, the membrane’s restoring force becomes insufficient to counteract the electrostatic force, leading to membrane collapse and loss of normal vibration capability. The resonant frequency is the intrinsic frequency at which the CMUT membrane achieves maximum vibration amplitude. By adjusting the driving signal frequency to match this resonance, the membrane’s vibrational response can be maximized, resulting in higher acoustic pressure output and improved echo signal quality. Proper tuning of the driving signal frequency is essential for optimizing CMUT transmission and reception performance.

Under ideal conditions where external loads are neglected, the CMUT undergoes simple harmonic motion under AC voltage excitation, with the dominant forces being electrostatic attraction and elastic restoring force. To simplify the modeling, the elastic restoring force due to structural deformation is represented by an ideal spring with stiffness $k_{0}$ and the distributed mass is approximated as a lumped mass. The capacitance is used to characterize the electrical properties of the CMUT, forming a mass–spring system model. This simplified model enables the analysis of key parameters such as the collapse voltage of the CMUT [29]. The net force $F_{N}$ acting on the CMUT is given by Equation (1):

(1) $$F_{N}=k_{0}x-\frac{\varepsilon_{0}SV_{dc}^{2}}{2{\left(g_{0}-x\right)}^{2}}$$

where $V_{dc}$ represents the voltage across the capacitor, $\varepsilon_{0}$ denotes the permittivity of vacuum, S is the membrane area, $g_{0}$ represents the initial cavity height, and $k_{0}$ represents the spring constant. Under static equilibrium, the net force satisfies $F_{N}$, from which the expression for the DC bias voltage $V_{dc}$ as a function of membrane displacement is derived. By taking the first derivative of $V_{dc}$ with respect to displacement and evaluating it at x=0, the system’s critical point can be determined. The analysis shows that collapse occurs when the membrane displacement reaches one-third of the cavity height, and substituting this value into the derived equation yields the collapse voltage, as given by Equation (2):

(2) $$V_{collapse}=\sqrt{\frac{8k_{0}g_{0}^{3}}{27\varepsilon_{0}S}}$$

where $V_{collapse}$ represents the collapse voltage of the CMUT. The fundamental frequency of a circular membrane, in the absence of external load and initial stress [30], is given by Equation (3):

(3) $$f_{c}=\frac{10.21}{2\pi R^{2}}\sqrt{\frac{D}{\rho h}}$$

where $f_{c}$ represents the first-order resonance frequency of the circular membrane, R denotes the membrane radius, D is the bending stiffness, ρ represents the membrane density, and h is the membrane thickness. The bending stiffness is determined by the material properties, and its calculation formula is given by Equation (4):

(4) $$D=\frac{\left(E+T\right)h^{3}}{12\left(1-v^{2}\right)}$$

where E represents the Young’s modulus; T denotes the surface tension, which is zero in the ideal case; v is the Poisson’s ratio; and h is the equivalent thickness of the multilayer membrane.

### 2.2. Finite Element Simulation Analysis

#### 2.2.1. Collapse Voltage

In this study, the collapse voltage of the CMUT is analyzed using steady-state simulations in COMSOL 6.1. A DC voltage sweep is applied to the top electrode, with the potential increasing in the direction toward the bottom electrode, causing the membrane to deflect vertically downward. By gradually increasing the DC bias, the critical threshold at which the membrane becomes unstable or abruptly displaces is identified as the collapse voltage. In the simulation, each DC bias voltage is applied independently, and the membrane displacement is recorded only after the system reaches steady-state equilibrium, thereby minimizing the influence of potential hysteresis effects.

**Figure 5:**
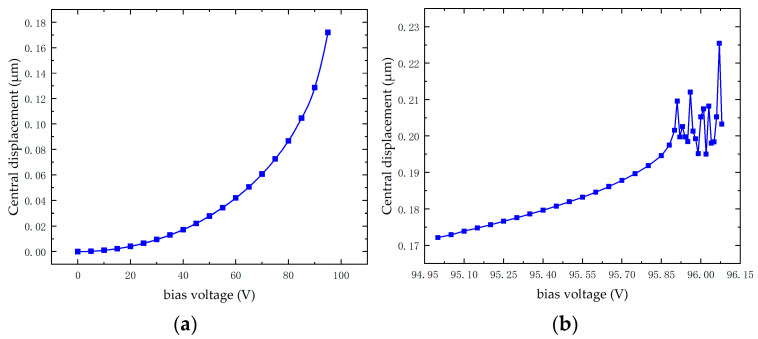
Simulation results of CMUT collapse voltage. (**a**) Graph of central displacement variation with bias voltage. (**b**) Bias voltage approaching collapse voltage.

According to the Figure 5, under full electrode coverage, the central displacement increases with the bias voltage, and its growth rate gradually rises. Theoretically, when the central displacement reaches 0.133 μm, the membrane enters the collapse state, corresponding to a bias voltage of 90.22 V. As the bias voltage further increases to 95.91 V, the central displacement undergoes a sudden change. Beyond this point, further increasing the voltage results in computational errors, indicating that the CMUT has reached its collapse voltage. In summary, the theoretical calculations closely align with the simulation results, verifying the accuracy of the model.

#### 2.2.2. Resonant Frequency

This paper utilizes the COMSOL frequency-domain analysis method to investigate the resonance characteristics of the CMUT. By performing frequency sweeps on impedance curves under various bias voltages and media, the center operating frequency of the CMUT is determined.

**Figure 6:**
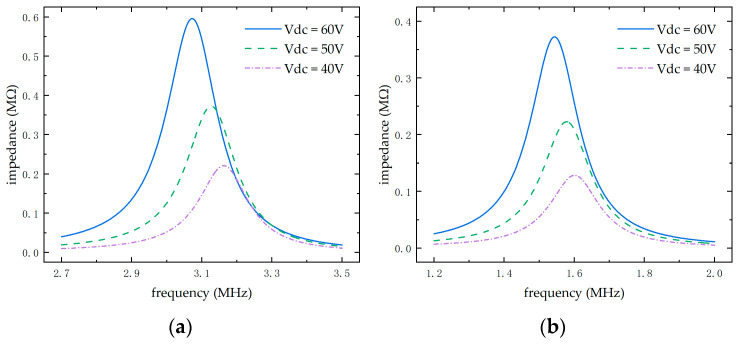
CMUT impedance curves in different media. (**a**) CMUT impedance variation curve in air. (**b**) CMUT impedance variation curve in water.

From the Figure 6 above, it can be seen that the medium in which the CMUT operates affects its central working frequency. Under a 40 V bias, the CMUT’s central frequency is 3.16 MHz in air but drops to 1.59 MHz in water due to the inertial load of water suppressing membrane vibration, effectively increasing system damping and shifting the frequency. Additionally, the bias voltage influences the central frequency; as the voltage increases, the impedance curve shifts left, and the membrane’s vibration amplitude increases, significantly enhancing CMUT performance within the collapse voltage range. The above results indicate that the finite element simulation results align with the theoretical derivation of the first-order resonance frequency.

## 3. Design of Drive and Detection System

### 3.1. System Overview

**Figure 7:**
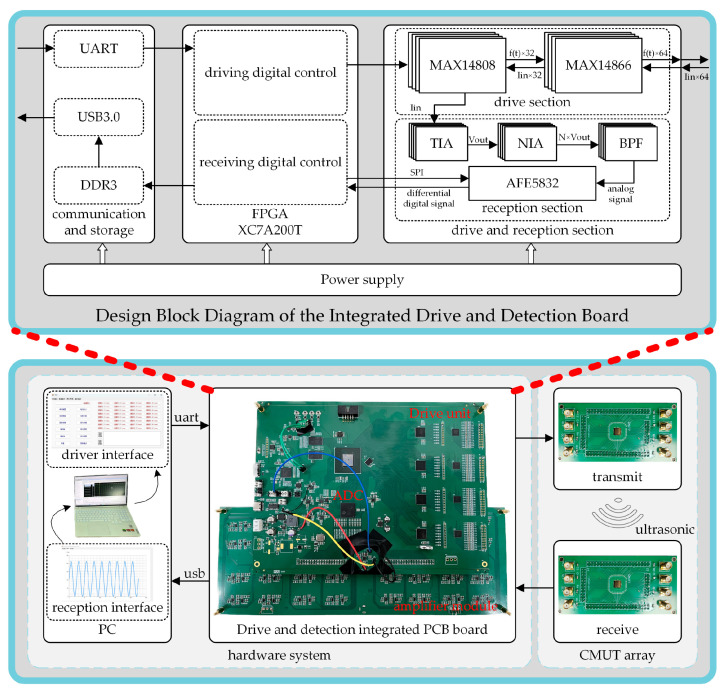
Drive and detection integrated system for CMUT array.

As shown in Figure 7, the CMUT array drive and detection integrated system is implemented based on a PC+FPGA architecture, comprising the hardware, software, and CMUT array ultrasonic transducer components. Firstly, The CMUT array ultrasonic transducer is responsible for transmitting and receiving ultrasound signals. Secondly, the hardware system, with the FPGA serving as the control core, includes modules for generating positive and negative pulse excitation signals, two-stage amplification of echo signals, 32-channel analog-to-digital conversion, data communication and storage, along with the personal computer (PC). In the end, the software part, implemented on the host computer, separately controls the excitation signals and performs digital signal post-processing. Compared with systems based on discrete control components, the proposed design enables 64-channel synchronous phase control. In addition, unlike conventional excitation modules, it allows for continuous excitation of the CMUT array to enhance the strength of the received echo signals. Furthermore, the system is capable of real-time acquisition of ultrasonic echo signals while simultaneously analyzing and adjusting the excitation waveform, aiming to achieve optimal echo performance.

### 3.2. Realization of Controllable Excitation Signal

#### 3.2.1. Focusing and Deflection of CMUT Array

**Figure 8:**
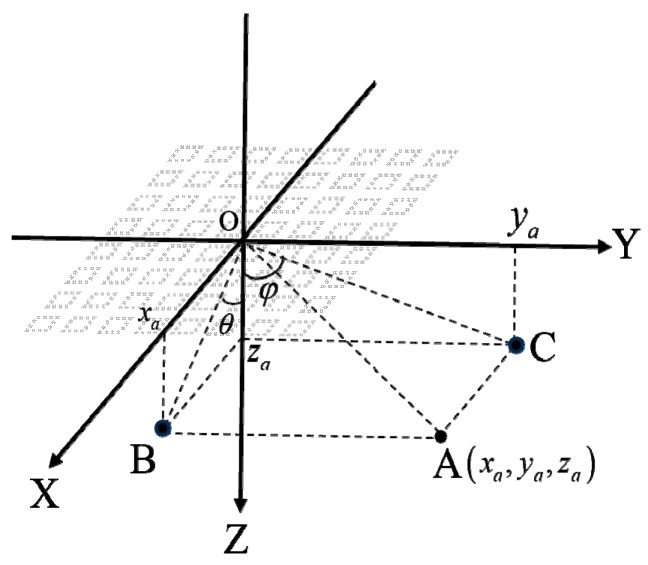
Schematic diagram of deflection and focusing of CMUT array.

Figure 8 illustrates a schematic of deflected focusing for the CMUT planar array. In the figure, point O represents the origin of the coordinate system, and point A is the focal point with a focal length of R. OB is the projection of OA onto the XOZ plane, while OC is the projection of OA onto the YOZ plane. The angle θ, defined as the angle between OB and the *Z*-axis, represents the lateral deflection angle of the focal point; the angle φ, defined as the angle between OC and the *Z*-axis, represents the vertical deflection angle.

Based on the description above, given the array element structural parameters, focal depth, and the corresponding deflection angles, the focal length can be calculated, as given by Equation (5):

(5) $$\left\{\begin{array}{c} R={\left(x_{a}^{2}+y_{a}^{2}+z_{a}^{2}\right)}^{1/2}\\ x_{a}=z_{a}tan\theta \\ y_{a}=z_{a}tan\varphi \\ z_{a}={\left(\frac{R^{2}}{1+{tan}^{2}\theta +{tan}^{2}\varphi }\right)}^{1/2} \end{array}\right.$$

At this point, let the coordinates of a given array element be $P\left(x_{ij},y_{ij},z_{ij}\right)$, and take the center of the phased array as the reference point. Then, the delay time expression for each array element can be derived, as given by Equation (6):

(6) $$∆t_{ij}=\frac{{\left[{\left(x_{ij}-x_{a}\right)}^{2}+{\left(y_{ij}-y_{a}\right)}^{2}+{\left(z_{ij}-z_{a}\right)}^{2}\right]}^{1/2}}{c}-\frac{R}{c}$$

where c represents the sound speed in the medium.

Overall, the above computation method is embedded in the host computer software, with the calculation precision retained up to 1 ns. The computed delay parameters for each channel are then packaged and transmitted via the serial port to the FPGA for subsequent digital processing.

#### 3.2.2. Real-Time Controllable Pulse Wave

The system designed in this paper achieves a drive delay precision of 1 ns, doubling the accuracy compared to conventional delay methods.

**Figure 9:**
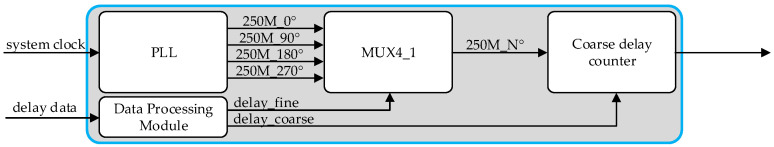
Schematic diagram of high-precision delay implementation.

The specific implementation is shown in Figure 9. First, the FPGA’s internal PLL is used to convert the 50 MHz system clock into four 250 MHz clock signals with a 90° phase difference. In this configuration, the relative phase shift between the four clock signals is 1 ns, while the period of the 250 MHz clock is 4 ns. Then, by selecting the appropriate clock using a 4-to-1 selector as the delay counting clock, a delay precision of 1 ns is achieved. The calculations of the coarse delay and fine delay are given by Equation (7).

(7) $$\left\{\begin{array}{c} delay_{fine}=t\;\mathrm{mod}\;4\\ delay_{coarse}=\left[\frac{t}{4}\right] \end{array}\right.$$

**Figure 10:**
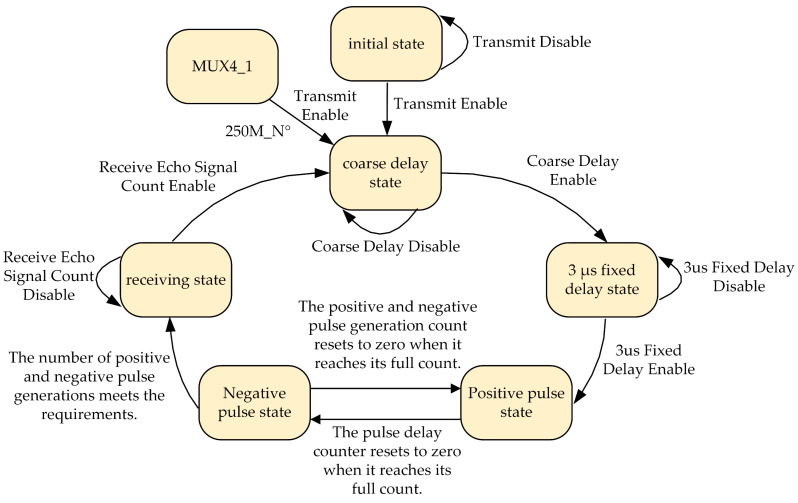
State transition flowchart of high-precision delay implementation.

Since the CMUT exhibits different center operating frequencies under varying media and bias voltages, and its excitation and echo signals are relatively weak, the system is designed to use positive and negative pulse signals with real-time adjustable excitation frequency and duration to enhance the signal-to-noise ratio (SNR) of the CMUT. The implementation involves transmitting data from the host computer to update specific RAM parameters within the FPGA in real time, while a state machine is used to write Verilog control code. The detailed state transition diagram is shown in Figure 10.

According to the state transition diagram, after configuring the differential control signal input pins of the MAX14808, the system drive module ultimately achieves 32-channel positive and negative pulse excitation signals. The fixed 3 µs delay is required by the MAX14808 datasheet, which helps reduce leakage current. Next, by configuring the internal registers of the MAX14866 chip via SPI, the ±30 V 32-channel excitation signals can be expanded to 64 channels. The adjustable parameters of the excitation signal include a frequency range from 10 kHz to 6.55 MHz, a pulse duration of 1 to 16 signal cycles, an excitation interval from 8 µs to 10 ms, and a programmable delay with a precision of 1 ns.

### 3.3. Circuit for Detecting High-Frequency Weak and Tiny Signals

**Figure 11:**
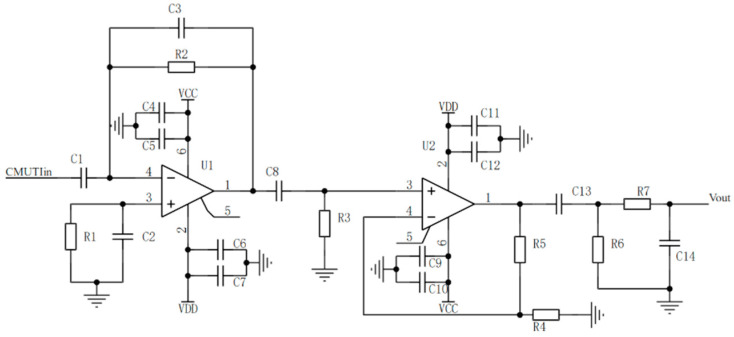
A two-stage amplifier circuit with a band-pass filter.

The planar CMUT discussed in this paper produces echo signals at the nA level and in the MHz frequency range, categorizing them as high-frequency weak signals. To meet the detection requirements of the AFE5832 multi-channel ADC chip, the current signal is first amplified and converted into a voltage signal using a transimpedance amplifier circuit. The voltage signal is then further amplified to the mV level via an in-phase amplification circuit, ensuring it falls within the detectable range of the AFE5832 chip. Finally, the AFE5832 module converts the analog signal into a digital signal, providing a reliable input for subsequent data processing. The schematic diagram of a two-stage amplifier circuit with a band-pass filter is shown in Figure 11.

**Figure 12:**
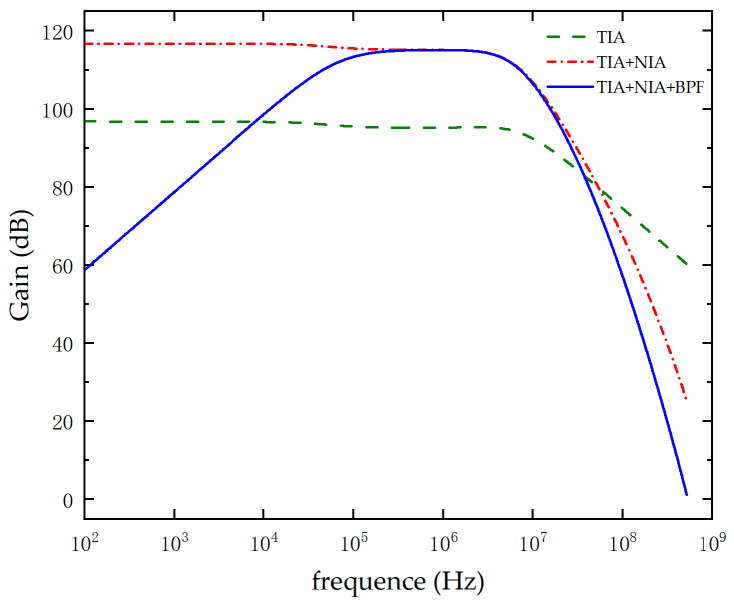
Bode plot of the gain for each stage in a two-stage amplifier circuit.

According to the above figure, the design of the first-stage transimpedance amplifier circuit must ensure minimal input bias current and offset current, minimal input noise, and a gain–bandwidth product that meets the detection requirements. The relevant calculations are shown in Equation (8):

(8) $$\left\{\begin{array}{c} f_{-3\mathrm{dB}}=\sqrt{\frac{f_{GBP}}{2\pi R_{2}C_{3}}}\\ i_{eq}=\sqrt{i_{B}^{2}+\frac{4KT}{R_{2}}+\frac{e_{n}^{2}}{R_{2}}+\frac{{\left(e_{N}2\pi FC_{3}\right)}^{2}}{3}} \end{array}\right.$$

where $f_{-3dB}$ represents the bandwidth, $f_{GBP}$ represents the gain–bandwidth product, $R_{2}$ is the feedback resistor, $C_{3}$ is the feedback capacitor used to eliminate system self-oscillation, $i_{eq}$ denotes the equivalent current noise, $i_{B}$ denotes the bias current at the inverting input, $e_{n}$ represents the voltage noise at the non-inverting input, and F represents the noise bandwidth. According to the above expression, $R_{2}$ decreases, the bandwidth increases, the gain decreases, and the noise increases. Therefore, an optimal balance between gain and bandwidth exists in the TIA circuit, necessitating the selection of appropriate parameters during the design process. Next, the second-stage in-phase amplification circuit, based on the virtual open-circuit principle, ensures that the input draws almost no current, thereby achieving high input impedance. Its low output impedance characteristic allows for the output signal to be fed directly into the AFE5832 module without significant attenuation. Finally, a band-pass filter is employed to suppress both high-frequency and low-frequency noise, thereby amplifying the high-frequency weak signal. A gain Bode plot of the second-stage amplification is obtained using a Cadence PSpice simulation model with appropriately optimized design parameters, as shown in Figure 12.

Based on the above figure, the −3 dB bandwidth of the two-stage amplifier circuit reaches 3.98 MHz, with an amplification gain of 113.72 dB. The passband range of the band-pass filter is 114.8 kHz to 3.89 MHz. The design of the two-stage amplifier circuit meets the requirements for the CMUT center operating frequency and amplification gain.

**Figure 13:**
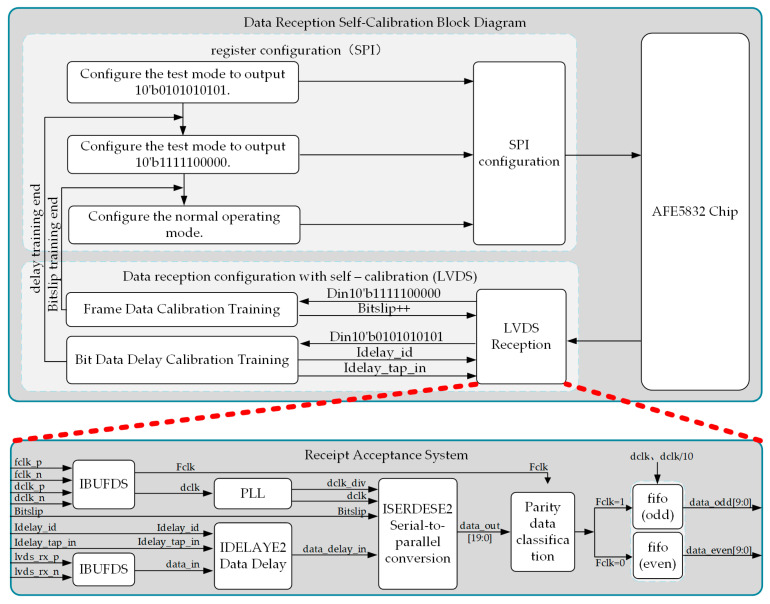
Block diagram of the implementation of the data self-calibration algorithm.

Figure 13 shows the design block diagram of the data LVDS reception self-calibration algorithm. First, in the Vivado2018.3 environment, the logic design for data LVDS reception is implemented using primitives, including IBUFDS (for converting differential signals to single-ended signals), IDELAYE2 (data delay module), ISERDESE2 (serial-to-parallel conversion module), and a data classification and readout module. Next, based on the received data, the built-in test mode of the AFE5832 chip is flexibly configured via SPI to achieve self-adjustment of the delay parameters and bit calibration parameters during LVDS reception, thereby significantly enhancing data reception accuracy. Finally, the system is capable of sampling 32-channel analog signals with a sampling rate of up to 50 Mbps and a data width of 10 bits.

## 4. Experimental Verification

### 4.1. Experimental Environment

**Figure 14:**
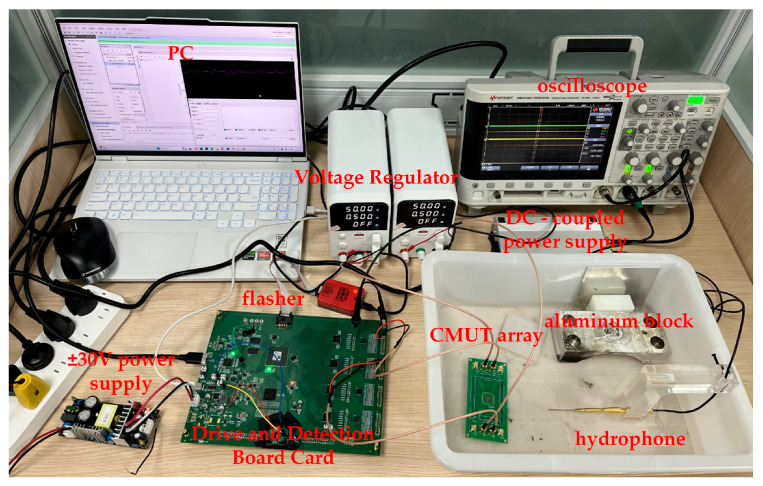
Diagram of the experimental environment.

This chapter mainly tests the designed CMUT-based drive and detection system. Figure 14 illustrates the experimental setup, which includes an oscilloscope (Keysight Technologies Inc., Santa Rosa, CA, USA), personal computer (PC), regulated power supply, ±30 V DC power supply, integrated drive and detection board, CMUT array, aluminum block, nh1000 hydrophone (Precision Acoustics Ltd., Dorchester, Dorset, UK), and other equipment. The tests specifically cover system excitation signal evaluation, integrated CMUT transmit–receive testing, and subsequent data post-processing. Experimental validation confirms that high-quality echo signals are obtained, demonstrating that the system has successfully implemented arrayed driving and echo signal detection for the CMUT, and that the resulting signals are suitable for ultrasonic imaging.

### 4.2. Performance Testing

#### 4.2.1. CMUT Impedance Testing

**Figure 15:**
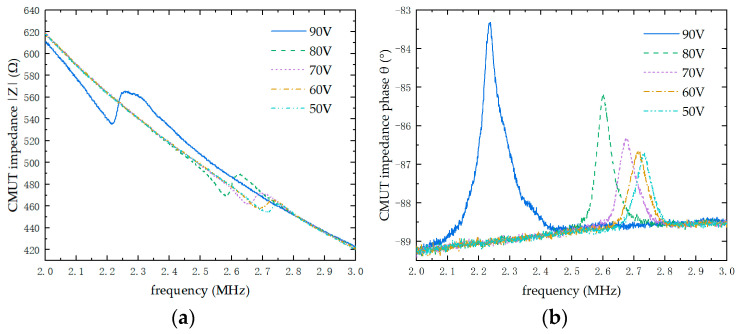
CMUT test results. (**a**) CMUT impedance curve. (**b**) CMUT phase curve.

Impedance and impedance phase are key indicators of the performance of a CMUT array ultrasonic transducer. The top and bottom electrodes of the CMUT are connected via gold wires and interfaced with an impedance analyzer for measurement. Meanwhile, a bias voltage ranging from 50 V to 90 V, with 10 V increments, is applied, and a frequency scan is conducted over the range from 2 MHz to 3 MHz. The resulting impedance and phase curves under different bias voltages in air are shown in Figure 15.

#### 4.2.2. LDV Characterization of the CMUT

By analyzing the impedance curves of the CMUT under different bias voltages, the frequency response range at each voltage level can be determined, which helps define the effective operating range of the device. While impedance and phase curves reveal the intrinsic properties of the CMUT, its actual vibrational behavior during operation must be validated through experimental testing. In this study, the dynamic motion of the CMUT under various bias voltages and excitation frequencies was characterized using a Lyncée Tec digital holographic microscope (DHM) from Switzerland.

**Figure 16:**
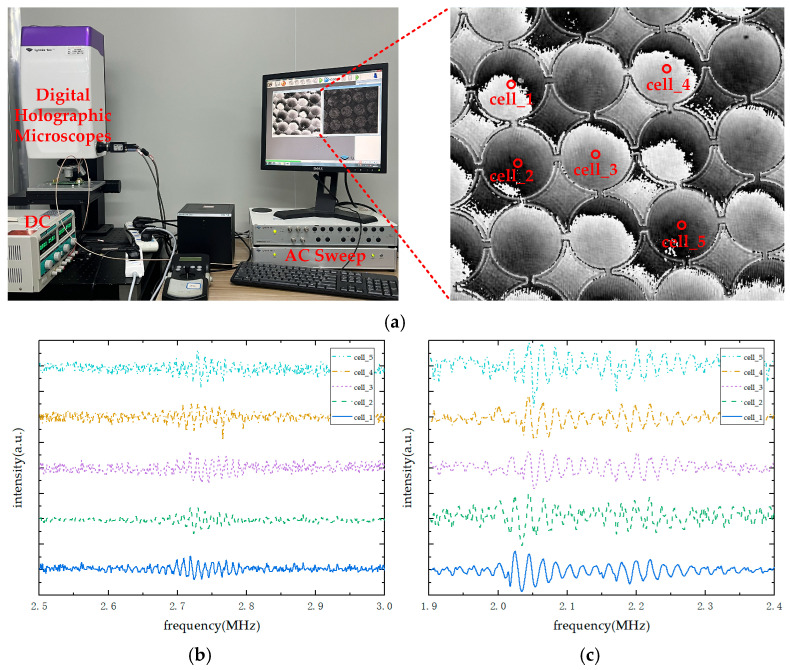
LDV characterization setup and measurement results for CMUT (**a**) LDV characterization setup and measurement results for CMUT. (**b**) Frequency response of CMUT center displacement under 50 V DC bias and ±2.5 V AC excitation. (**c**) Frequency response of CMUT center displacement under 90 V DC bias and ±2.5 V AC excitation.

Figure 16a shows the experimental setup for CMUT vibration characterization. The CMUT chip was first securely mounted under the digital holographic microscope. After calibrating the reference plane using the associated software and ensuring the chip remained stable, a DC bias voltage and an AC excitation were applied. An AC frequency sweep was then performed to capture the full-surface vibration response of the CMUT. The recorded data were reconstructed, and the displacement at the center of selected CMUT cells was extracted, yielding vibration results for multiple CMUT units.

Figure 16b,c show the center displacement of five groups of CMUT cells under bias voltages of 50 V and 90 V, respectively. It is clearly observed that the CMUT’s center operating frequency shifts to a lower value, and the vibration amplitude increases significantly at the higher bias voltage. These results further confirm that increasing the bias voltage notably enhances CMUT performance. Moreover, the vibration test results are highly consistent with those obtained from the impedance analyzer, validating the fundamental characteristics of the CMUT.

Therefore, the driving signal of the CMUT ultrasonic driving and detection system is required to be able to generate an excitation signal with an adjustable frequency, and the frequency of the excitation signal needs to reach above 2.8 MHz. Next, the hardware driving system is tested to verify its driving function.

#### 4.2.3. Drive Testing

**Figure 17:**
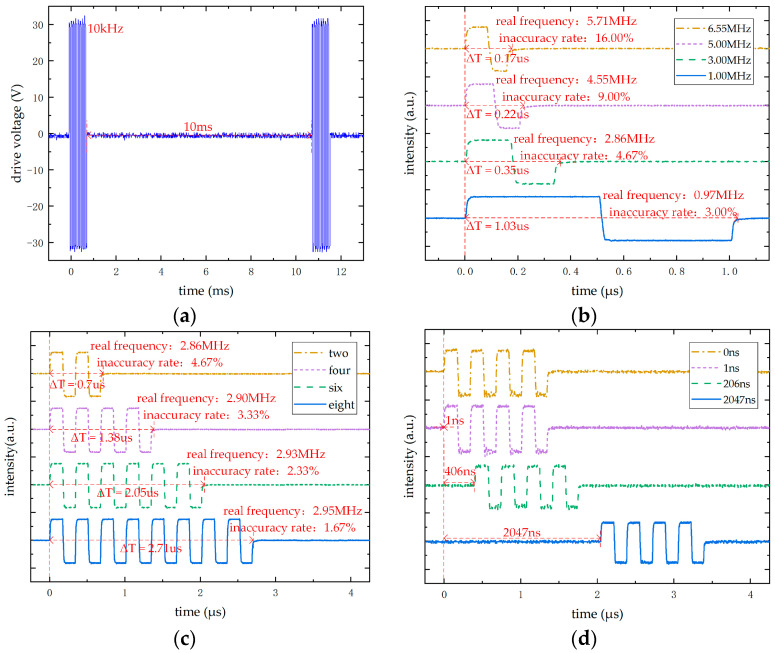
Test results of the driving signal. (**a**) The single-cycle excitation signal. (**b**) CMUT excitation signals at different frequencies. (**c**) CMUT excitation signals with different excitation durations. (**d**) CMUT excitation signals under different delay configurations.

This subsection presents the characterization of driving excitation signals for the system design. The tests include inter-pulse interval characterization, frequency adjustment range characterization, period adjustment range characterization, delay adjustment range characterization, and delay adjustment precision characterization. Testing was conducted using a hardware system board, PC-based host computer, oscilloscope, and other equipment. Test results are shown in Figure 17.

Figure 17a presents the test results of the single ultrasonic excitation signal, which is executed repeatedly. The signal parameters are as follows: an amplitude of ±30 V, a frequency of 10 kHz, an excitation duration of eight positive and negative pulse cycles, and an excitation interval of 10 ms. This excitation method not only compensates for the potential insufficient driving capability caused by single-pulse excitation during CMUT operation but also avoids damage resulting from severe contact between the upper and lower electrodes due to long-term continuous excitation. Additionally, it prevents the MAX14808 chip from overheating or even bursting caused by continuous generation of high-voltage positive and negative pulse excitation signals.

Figure 17b illustrates the single positive and negative pulse waves under different frequency configurations. The configured frequency parameters are 1 MHz, 3 MHz, 5 MHz, and the maximum frequency of 6.55 MHz, with a resolution of 100 Hz. As shown in the figure, the adjustable frequency range meets the operational requirements of the CMUT and allows for the frequency to be adjusted continuously within this range. However, the detected output frequency is slightly lower than the preconfigured value, and the frequency error increases as the preconfigured frequency rises. This discrepancy is primarily attributed to the high-speed performance limitations of the FPGA and the setup time of the MAX14808 output signal. Therefore, in practical applications, it is essential to measure and calibrate the output frequency of the driving signal to ensure accuracy.

Figure 17c presents the driving signals with different excitation durations, set to two, four, six, and eight pulse cycles at a frequency of 3 MHz. Combined with Figure 16b, the tunable excitation duration ranges from one to eight pulse cycles. As the excitation duration increases, on one hand, the echo signal intensity of the CMUT increases; on the other hand, the influence of the signal establishment time on the excitation signal frequency decreases, thereby reducing the frequency error. Additionally, the bandwidth of the CMUT echo signal increases, which is beneficial for improving the quality of the echo signal compared to a single-pulse excitation method. However, as the excitation duration increases, the duration of the echo signal also lengthens, potentially leading to imaging artifacts. Therefore, appropriately selecting the excitation pulse duration can significantly enhance the system’s detection performance.

Figure 17d shows the driving signals under different delay configurations. From the figure, the excitation delays are set to 0 ns, 1 ns, 406 ns, and a maximum delay of 2047 ns. The driving signal has a duration of four pulse cycles at 3 MHz. The aforementioned four delay configuration parameters correspond to fine delay clocks of 0 ns, 1 ns, 2 ns, and 3 ns, respectively, which verifies the high-precision delay algorithm implemented via PLL phase shifting. Moreover, the maximum delay of 2047 ns meet the delay requirements of the CMUT array, and each coarse delay parameter occupies only a 9-bit register, optimizing FPGA resource utilization. In conclusion, the current delay parameter configuration successfully meets the system’s requirements for focal steering and beamforming.

#### 4.2.4. System Testing

The system testing is mainly divided into the system transmission performance test based on CMUT and the integrated transmitting and receiving test based on CMUT. The schematic diagram of the testing method is shown in Figure 17.

**Figure 18:**
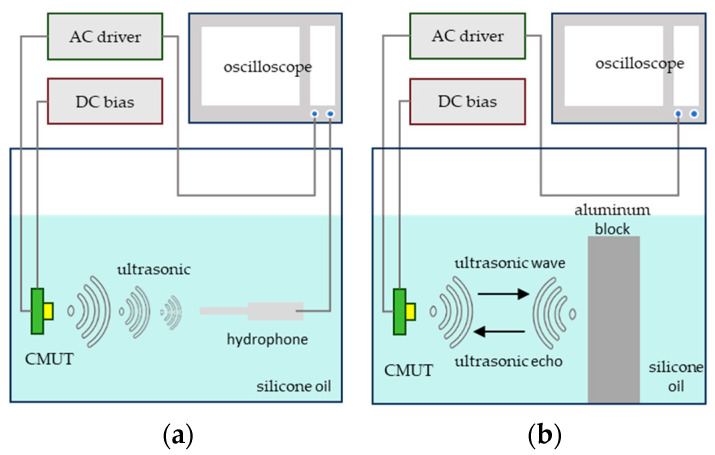
Schematic diagram of the testing method. (**a**) Transmission performance test of CMUT. (**b**) Integrated transmitting and receiving test of CMUT.

**Figure 19:**
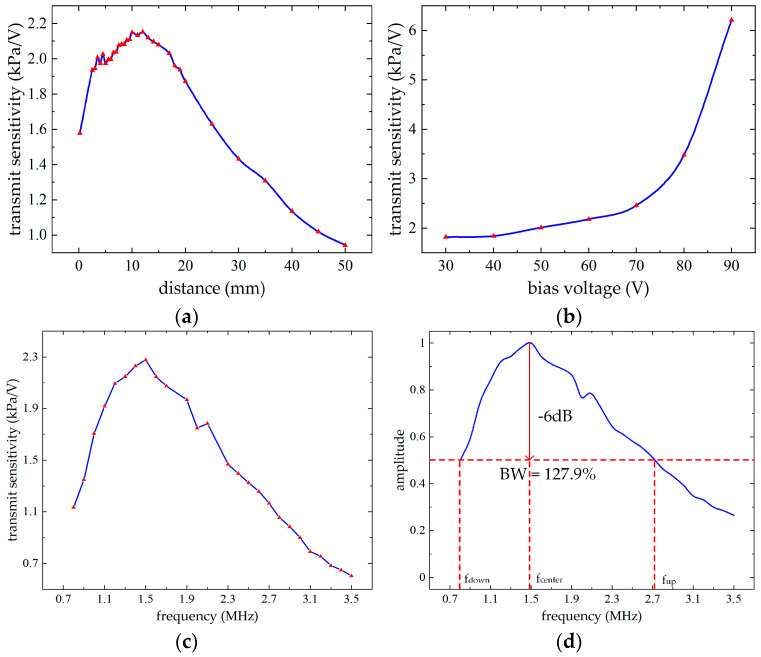
Results of the transmitting sensitivity of CMUT. (**a**) The test results of the transmitting sensitivity with the transmitting distance. (**b**) The test results of the transmitting sensitivity with the voltage. (**c**) The test results of the transmitting sensitivity varying with frequency. (**d**) The analysis results of the −6 dB bandwidth of the CMUT.

Figure 18a shows the equipment required in the process of measuring the transmitting sensitivity, including the CMUT, the hardware driving system, the DC bias power supply, the hydrophone, and the oscilloscope. In the specific implementation process, the voltage of the ultrasonic waves emitted by the CMUT is measured by the hydrophone, and the transmitting sound pressure intensity of the CMUT is calibrated using the receiving sensitivity table of the hydrophone. Finally, the transmitting sensitivity of the CMUT array is calculated through the formula shown in Equation (9):

(9) $$S_{emit}=\frac{V_{rec}}{S_{rec}\cdot L\cdot V_{emit}}$$

where $V_{rec}$ represents the voltage received by the receiving hydrophone, L represents the distance between the hydrophone and the surface of the CMUT, and $S_{rec}$ represents the receiving sensitivity of the hydrophone. The performance of the CMUT is reflected by the variation of the transmitting sensitivity. The test results of the transmitting sensitivity varying with distance, bias voltage, and excitation signal frequency are shown are shown in Figure 19.

As shown in Figure 19a, the transmitting sensitivity of the CMUT array first increases and then decreases with distance within the range of 0.2 mm to 50 mm. The sensitivity exhibits relatively small variations between 6.5 mm and 17 mm due to the optimal effect of acoustic beam superposition within the focused region along the normal direction. The maximum transmitting sensitivity reaches 2.1519 kPa/V at a distance of 12 mm, corresponding to an AC driving frequency of 1.6 MHz and a DC bias of 60 V.

As shown in Figure 19b, the transmitting sensitivity increases gradually with voltage, with an accelerating rate of change, consistent with theoretical and simulation analyses. Under a bias voltage range of 30 V to 90 V, the minimum transmitting sensitivity is 1.8208 kPa/V and the maximum reaches 6.2132 kPa/V. These results correspond to a transmitting distance of 10 mm and a frequency of 1.6 MHz. To ensure stable CMUT performance, a bias voltage between 50 V and 60 V is typically selected for operation.

As shown in Figure 19c, when operating in silicone oil, the transmitting sensitivity of the CMUT first increases and then decreases with frequency within the range of 0.8 MHz to 3.5 MHz, which is consistent with the operational behavior under the first-order vibration mode. The maximum transmitting sensitivity reaches 2.2778 kPa/V in the figure, corresponding to an operating frequency of 1.5 MHz and a DC bias voltage of 50 V.

Normalization of the test data in Figure 19c yields the results shown in Figure 19d. At a driving signal frequency of 0.802 MHz and an operating frequency of 2.72 MHz, the transmitting sensitivity corresponds to 0.501 times the maximum value. The calculated center frequency is approximately 1.4995 MHz, with a relative bandwidth of 127.9%@−6 dB. It is observed that the experimentally measured center frequency of the CMUT in silicone oil is 1.4995 MHz, which is significantly lower than the simulated value of 3.16 MHz in air. This discrepancy is primarily attributed to the acoustic loading effect of the liquid medium. Silicone oil, having higher density and acoustic impedance than air, introduces additional damping and increases the effective mass of the vibrating membrane. As a result, the resonant frequency of the CMUT shifts leftward, which is consistent with the theoretical understanding of CMUT behavior in liquid environments. Overall, the CMUT performance meets the ultrasonic imaging requirements.

**Figure 20:**
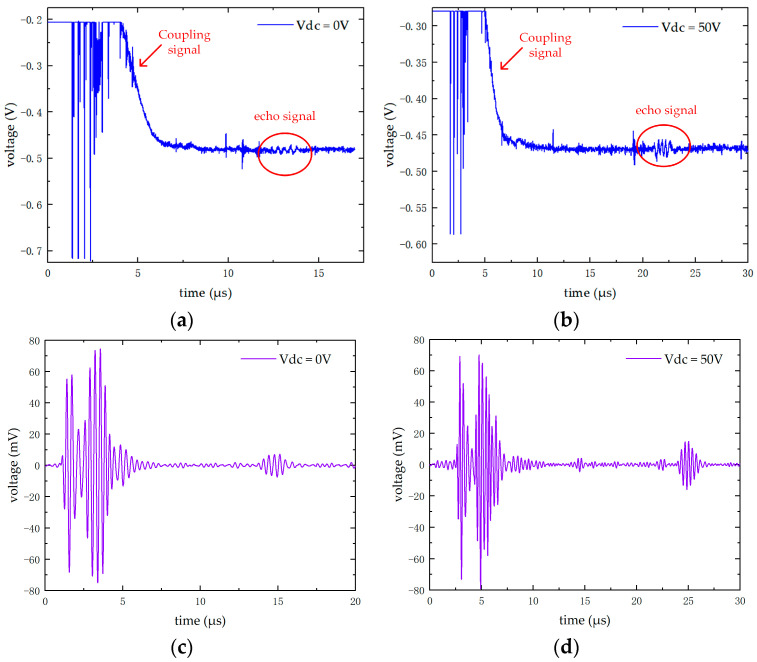
The test results of the integrated transmitting and receiving test. (**a**) The test results without bias voltage. (**b**) The test results with a bias voltage of 50 V. (**c**) The results of digital filtering processing of the echo without bias voltage. (**d**) The results of digital filtering processing of the echo with a bias voltage of 50 V.

Figure 20 shows the test results of the integrated transmitting and receiving experiment. Without bias voltage, the time interval between the first echo and the coupling signal is 14.2 μs. Through calculation, the distance between the CMUT and the aluminum block is 10.9 mm. At this time, the peak-to-peak value of the corresponding signal is 14 mV, and the frequency is 3 MHz. When a bias voltage with a magnitude of 50 V is applied, the time interval between the first echo and the coupling signal is 22.3 μs. Calculation shows that the CMUT is 17.2 mm away from the aluminum block. The peak-to-peak value of the corresponding signal is 29.25 mV and the frequency is 3 MHz. Based on this, the system function is preliminarily verified, and the conclusion that the bias voltage significantly improves the CMUT performance is obtained. Meanwhile, the echo data obtained from the experiment are processed by an eighth-order Butterworth digital filter implemented using four amplifier stages, with a cutoff frequency range from 2.29 MHz to 3.9 MHz. A digital signal suitable for imaging is thus obtained, verifying that the CMUT-based driving and detection system designed in this paper can be applied to subsequent imaging tasks.

## 5. Conclusions

To overcome the shortcomings of ultrasonic imaging systems based on piezoelectric transducers, such as large size, low array integration, and significant imaging artifacts, this paper proposes an ultrasonic driving and detection system based on an 8 × 8 planar CMUT array. The system utilizes a single board with an area of only 480 cm^2^ to drive a 64-channel CMUT and to perform analog acquisition of 32-channel ultrasonic echo signals. The specific implementation parameters include an excitation signal frequency range of 10 kHz to 5.71 MHz, an excitation duration of up to eight pulse cycles, precise phase control with a delay accuracy of 1 ns, an ADC sampling rate of 50 Mbps, and a data width of 10 bits. During system implementation, to address the issue of weak current signals generated by the CMUT, each element of the CMUT probe is composed of 6 × 6 CMUT units on the transmission side, thereby enhancing the transmitted signal; on the echo reception side, a two-stage amplification circuit is designed to improve signal quality, with key parameters of a −3 dB bandwidth of 3.89 MHz and a gain of up to 113.72 dB. Experimental validation demonstrates that the MEMS-based CMUT array driving and detection system achieves the intended performance and is suitable for ultrasonic imaging.

## Figures and Tables

**Table 1:** CMUT structural parameters.

Structural Name	Thickness/μm
Top electrode	0.2
Top insulation layer	0.3
Membrane	2
Vacuum cavity	0.4
Edge support	0.4
Bottom insulation layer	0.15

**Table 2:** CMUT array structural parameters.

Structural Name	Value/μm
cells spacing/$d_{c}$	100
cells gap/$g_{c}$	10
array element diameter/*L*	590
array element spacing/$d_{e}$	620
array element gap/$g_{e}$	30
aperture/*A*	4930

## Data Availability

The original contributions presented in this study are included in the article. Further inquiries can be directed to the corresponding authors.

## References

[1] Shi K., Guo Y. (2010). Phased Array Ultrasonic Imaging and Testing.

[2] Dew E.B., Zemp R.J. (2023). High-Performance Electrode-Post CMUTs: Fabrication Details and Best Practices. IEEE Trans. Ultrason. Ferroelectr. Freq. Control.

[3] Wang H., Yang H., Chen Z., Zheng Q., Jiang H., Feng P.X.L., Xie H. (2021). Development of dual-frequency PMUT arrays based on thin ceramic PZT for endoscopic photoacoustic imaging. J. Microelectromech. Syst..

[4] Moisello E., Novaresi L., Sarkar E., Malcovati P., Costa T., Bonizzoni E. (2024). PMUT and CMUT devices for biomedical applications: A review. IEEE Access.

[5] Ding X., Wu Z., Gao M., Chen M., Li J., Wu T., Lou L. (2022). A high-sensitivity bowel sound electronic monitor based on piezoelectric micromachined ultrasonic transducers. Micromachines.

[6] Ding H., Yang D., Qu M., Yang C., Chen X., Le X., Zhu K., Xu J., Lin L., Xie J. (2021). A pulsed wave Doppler ultrasound blood flowmeter by PMUTs. J. Microelectromechanical Syst..

[7] Manwar R., Kratkiewicz K., Avanaki K. (2020). Overview of ultrasound detection technologies for photoacoustic imaging. Micromachines.

[8] Emadi T.A., Buchanan D.A. (2015). A novel 6× 6 element MEMS capacitive ultrasonic transducer with multiple moving membranes for high performance imaging applications. Sens. Actuators A Phys..

[9] Maadi M., Ceroici C., Zemp R.J. (2021). Dual-frequency CMUT arrays for multiband ultrasound imaging applications. IEEE Trans. Ultrason. Ferroelectr. Freq. Control.

[10] Joseph J., Ma B., Khuri-Yakub B.T. (2021). Applications of capacitive micromachined ultrasonic transducers: A comprehensive review. IEEE Trans. Ultrason. Ferroelectr. Freq. Control.

[11] Yoon H.S., Chang C., Jang J.H., Bhuyan A., Choe J.W., Nikoozadeh A., Watkins R.D., Stephens D.N., Pauly K.B., Khuri-Yakub B.T. (2016). Ex Vivo HIFU Experiments Using a 32×32-Element CMUT Array. A comprehensive review. IEEE Trans. Ultrason. Ferroelectr. Freq. Control.

[12] Suarez-Castellanos I.M., de Sallmard G., Vanstaevel G., Ganeau A., Bawiec C., Chapelon J.Y., Guillen N., Sénégond N., N’Djin W.A. (2023). Dynamic ultrasound focusing and centimeter-scale ex vivo tissue ablations with a CMUT probe developed for endocavitary HIFU therapies. IEEE Trans. Ultrason. Ferroelectr. Freq. Control.

[13] Gholampour A., Muller J.W., Cano C., van Sambeek M.R., Lopata R., Schwab H.M., Wu M. (2023). Multiperspective photoacoustic imaging using spatially diverse CMUTs. IEEE Trans. Ultrason. Ferroelectr. Freq. Control.

[14] Ghavami M., Sobhani M.R., Zemp R. (2023). Transparent dual-frequency CMUT arrays for photoacoustic imaging. IEEE Trans. Ultrason. Ferroelectr. Freq. Control.

[15] Wong S.H., Kupnik M., Zhuang X., Lin D.S., Butts-Pauly K., Khuri-Yakub B.T. (2008). Evaluation of wafer bonded CMUTs with rectangular membranes featuring high fill factor. IEEE Trans. Ultrason. Ferroelectr. Freq. Control.

[16] Cheng X., Chen J., Shen I.M., Li P.C., Wang M.H. 6F-3 fabrication and assembly of a monolithic 3D CMUT array for imaging applications. Proceedings of the 2008 IEEE Ultrasonics Symposium.

[17] Na S., Li Z., Wong L.L., Chen A.I.H., Macecek M., Yeow J.T. (2017). An optimization and comparative study of air-coupled CMUT cells with circular and annular geometries. IEEE Trans. Ultrason. Ferroelectr. Freq. Control.

[18] Maadi M., Zemp R.J. (2019). A nonlinear lumped equivalent circuit model for a single uncollapsed square CMUT cell. IEEE Trans. Ultrason. Ferroelectr. Freq. Control..

[19] Huang X., Wang H., Yu L. (2021). Investigation on design theory and performance analysis of vacuum capacitive micromachined ultrasonic transducer. Micromachines.

[20] Li Z., Zhao L., Zhao Y., Li J., Xu T., Hu K., Liu Z., Yang P., Luo G., Lin Q. (2020). Closed-form expressions on CMUTs with layered anisotropic microplates under residual stress and pressure. IEEE Trans. Ultrason. Ferroelectr. Freq. Control.

[21] Pei Y., Zhang Y., Hu S., Wang Z., Li Y., He C., Zhang S., Wang R., Zhang W., Zhang G. (2021). Breast transmission ultrasound tomography based on capacitive micromachined ultrasonic transducer linear arrays. IEEE Sens. J..

[22] Adelegan O.J., Coutant Z.A., Zhang X., Yamaner F.Y., Oralkan Ö. (2020). Fabrication of 2D capacitive micromachined ultrasonic transducer (CMUT) arrays on insulating substrates with through-wafer interconnects using sacrificial release process. J. Microelectromech. Syst..

[23] Saccher M., Savoia A.S., Van Schaijk R., Klootwijk J.H., Dekker R. (2024). Pre-Charged Collapse-Mode Capacitive Micromachined Ultrasonic Transducers (CMUT) Receivers for Efficient Power Transfer. IEEE Trans. Ultrason. Ferroelectr. Freq. Control.

[24] Jia L., Liang Y., Meng F., Wang Z., Zhang G., Wang R., He C., Yang Y., Cui J., Zhang W. (2024). Research on Acoustic Impedance Matching Method of CMUT Sensor Based on PDMS/BN Particles. IEEE Sens. J..

[25] Farhanieh O., Sahafi A., Roy R.B., Ergun A.S., Bozkurt A. (2017). Integrated HIFU drive system on a chip for CMUT-based catheter ablation system. IEEE Trans. Biomed. Circuits Syst..

[26] Zhou M., Ouzounov S., Cantatore E., Harpe P. (2021). An RX AFE with programmable BP filter and digitization for ultrasound harmonic imaging. IEEE Trans. Biomed. Circuits Syst.

[27] Wang H., Wang X., He C., Xue C. (2019). Reception characteristics investigation and measurement of capacitive micromachined ultrasonic transducer. Sens. Rev..

[28] Maadi M., Chee R., Zemp R.J. Mutual radiation impedance for modeling of multi-frequency CMUT arrays. Proceedings of the 2015 IEEE International Ultrasonics Symposium (IUS).

[29] Zhao L., Jiang Z., Li Z., Zhao Y., Huang Q.A. (2018). Modeling of electrostatically actuated microplates. Micro Electro Mechanical Systems.

[30] Zhao Y., Zhao L., Li Z., Li J., Yang P., Xu T., Liu Z., Guo S., Wang J., Jiang Z. (2019). Capacitive micromachined ultrasonic transducers for transmitting and receiving ultrasound in air. J. Micromech. Microeng..

